# Predictive validity of a Uniform Entrance Test for the health professionals

**DOI:** 10.12669/pjms.35.2.334

**Published:** 2019

**Authors:** Rahila Ali, Syeda Kauser Ali, Azam Afzal

**Affiliations:** 1 *Rahila Ali, (RA): MCPS-FM, MRCGP (INT), MHPE. Department for Educational Development, Aga Khan University, Karachi, Pakistan*; 2 *Syeda Kauser Ali (SKA): MHPE, PhD. Department for Educational Development, Aga Khan University, Karachi, Pakistan*; 3 *Azam Afzal,(AA): MHPE. Department for Educational Development, Aga Khan University, Karachi, Pakistan*

**Keywords:** Student selection, Admission tests, Validity, Academic performance

## Abstract

**Objective:**

To investigate the predictive validity of Uniform Entrance Test for academic performance in the first two years in various health science degree programs.

**Methods:**

A retrospective analysis of admissions data and academic performance of students admitted in under-graduate programs of medicine, dentistry and pharmacy of three cohorts was taken. The independent and dependent variables were entry test scores and semester scores respectively. Spearman’s Correlation co-efficient was computed to determine the association between entrance test scores and semester scores for three groups.

**Results:**

Majority of the students were from the MBBS degree program (61%) with majority of female students (65%) in all three programs. In MBBS the highest correlation coefficient between entry test and semester scores was observed for semester one *rs* = 0.334 and lowest in semester four *rs*= 0.208. In BDS degree program both highest and lowest correlations were in semester one. In the Pharm-D degree program, a significant correlation was only seen in cohort 1 but not in the subsequent cohorts.

**Conclusion:**

The uniform entrance test has an incremental predictive validity for the MBBS and BDS programs as compared to Pharm-D. Better performance in the entrance test predicts higher semester scores and more likelihood of achieving higher scores in the first year as compared to the second year.

## INTRODUCTION

Admission in health professional colleges has always been competitive. In Pakistan there is an increasing number of applicants aspiring to seek medical, dental and pharmacy education.^1^ This is hand-in-hand with a rise in the number of institutes offering professional training. Programs aim to select applicants likely to complete the program requirements and demonstrate good academic performance.^2^

Commonly used criteria for admission include academic ability judged by prior academic achievements and entrance test scores; personality, motivation and communication skills are gauged by interviews; awareness of health care related professions demonstrated by voluntary work experiences and involvement in extracurricular activities.^3^

Previously, before the unitary concept of validity, validity was classified as three separate types: content, criterion and construct. Criterion-related validity was split into concurrent and predictive, subject on the timing of the data collection for the criterion evidence. Predictive validity is the degree to which test scores are able to predict future performance on a domain of interest.^4^ This was replaced by Kane’s unitary concept of validity for which evidence was collected. According to this contemporary framework the older “predictive validity” is the relationship of the test scores with other variables or “correlation with another measure having an expected relationship” such as test–criterion correlations. To elaborate, an important validity evidence for a test such as the Medical College Admission Test (MCAT) will be the predictive relationship between test scores and medical school achievement.^4^,^5^

Predictive validity of the selection instruments facilitates student selection^6^ and most of the studies from medicine, dentistry and pharmacy institutes establish a positive correlation between admission test scores (criterion measure) and academic achievements (outcome measure) during the first two years.^7^,^8^

MCAT has been reported to be a good predictor of academic performance in the first two (pre-clinical) years of medical school. A meta-analysis of weighted effects sizes (r) reported predictive validity coefficient for the MCAT in the preclinical years of r- 0.39^9^ predictive power of MCAT for Medical school GPA decreasing from 0.44 for first and second year to 0.32 in third year.^10^ Similar findings have been reported for Undergraduate Medical and Health Science Admission Test (UMAT),^11^ the Biomedical Admission Test (BMAT),^8^ and the Aptitude and Achievement test.^6^

Studies of predictive validity of Dental school Admission Tests (DAT) reported from different countries have shown moderate to weak positive correlation between DAT scores and academic performance in dental schools tending to decline from year one to year three.^12^-^14^

Pharmacy school admission tests (PCAT) scores used for admission to Pharmacy schools in the US optimally identify students likely to succeed (r = 0.35 - 0.77) in specific course work as well as throughout the course of the program.^15^-^17^

The Graduate Australian Medical School Admission Test (GAMSAT) used for selection to medical, dental and veterinary science programs is reported as a poor predictor of medical school performance.^18^,^19^ The Saudi National Achievement Examination used for admission in four different health care disciplines is reported as predictive of future performance in all disciplines, however there were significant differences between students of different health care disciplines with medical students performing better than their health science counterparts.^6^

The limited research available in Pakistan is largely restricted to medical college admissions and reports a weak to moderately positive correlation coefficient (r= 0.21- 0.4) in the initial years, which decreases as a student progresses.^7^,^20^

The selection of medical students admitted to Ziauddin University (ZU) was earlier based on their high school achievements, entrance test score and interview ratings. With introduction of doctor of Pharmacy (Pharm-D) and Bachelor of dental Sciences (BDS) the admissions committee has been offering a Uniform Entrance Test (UET) since 2010. An earlier study reported no significant relationship between the admission test scores and scores obtained in the professional examination of the Bachelor of Medicine and Bachelor of Surgery (MBBS).^21^

The objective of the study was to determine the predictive validity of the Uniform Entrance Test for academic performance in the first two years of various health science programs (medicine, dentistry and pharmacy) as demonstrated by scores on the first four semester examination.

## METHODS

This study was conducted at Ziauddin University (MBBS, BDS and Pham-D programs).Ethical approval was obtained from Ethical review Committee (ERC) of Ziauddin University.

### Inclusion and exclusion criteria

All candidates who sat the ‘Uniform Entrance Test’ of ZU from 2010 till 2012 (cohort 1-3) and completed at least four semesters of study were included. Those candidates who withdrew from the program before completion of two years, who were considered ineligible to sit for semester exam; repeated the semesters and took exam with next batch or had to take re-sit examinations were excluded. In case of repeaters, only their first score was considered. The sample consisted of data of 475 students from three cohorts of students admitted in the programs of MBBS, BDS and Pharm-D at ZU.

The independent variable included the entry test score while the the dependent variable comprised of the individual scores of theory and practical exam; and the aggregate semester score.

A correlational study design was used to study the predictive validity of UET for scores on theory and practical examinations of end of semester examination and the aggregate semester score.

### Statistical analysis

Data was analyzed using SPSS version 20. The statistically significant difference for means was set at *p<*0.05. The mean and Standard Deviation (SD) of entrance test score and semester result (theory, practical and grand total) were computed for each group of students. Since the data was not normally distributed, Spearman Rank correlation coefficient was used to assess the correlation between selection criterion (entry test) and academic achievement (theory, practical and aggregate semester score).

## RESULTS

Majority of the candidates (60%) were from MBBS, 23% from BDS and 17% from Pharm-D. In all three years and across all programs majority of the candidates were female. Approximately 247 (53%) students were from the Pakistani system of education while 221 (47%) from the British system of education (Cambridge International Examination -A-Levels).

**Fig.1:**
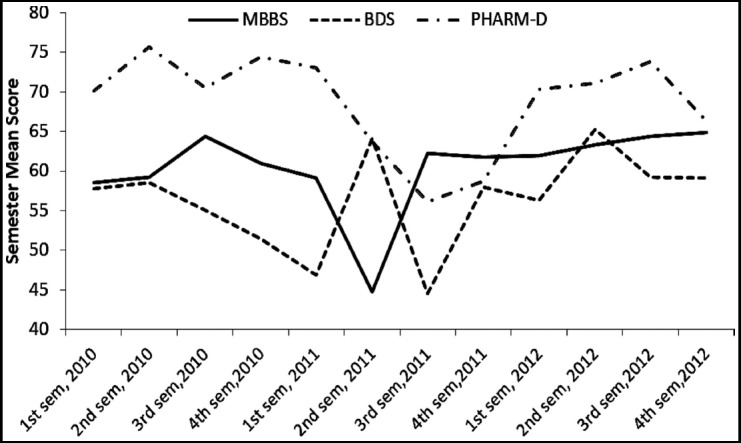
Mean semester score of MBBS, BDS and Pharm D (Cohort 1-3).

The means and standard deviation were computed (Table-I) and significant difference in the mean semester scores in all three programs of the three cohorts was found (Fig.1). The mean scores of Pharm-D differed significantly from the other two program; and remained higher throughout three years than the mean scores of the other two programs.

**Table-I:** Mean and Standard Deviation of student’s scores of the MBBS, BDS and Pharm -D programs.

MBBS				BDS			Pharm-D		

Exam	Mean±SD Cohort 1(2010)	Mean±SD Cohort 2 (2011)	Mean±SD Cohort 3(2012)	Mean±SD Cohort 1(2010)	Mean±SD Cohort 2 (2011)	Mean±SD Cohort 3(2012)	Mean±SD Cohort 1(2010)	Mean±SD Cohort 2 (2011)	Mean±SD Cohort 3(2012)
***1^st^ Semester***									
Theory	57.85±6.52	54.94±15.46	58.24±14.91	57.94±14.02	44.47±29.41	53.97±18.80	67.24±17.47	68.11±10.55	67.73±10.60
Practical	67.89±8.72	63.22±17.90	65.71±16.19	57.72±13.05	49.38±32.75	58.65±23.07	73.60±16.81	78.66±8.39	73.46±10.13
Grand Total	58.50±17.85	59.14±16.53	61.98±15.36	57.75±13.21	46.85±30.99	56.31±20.30	70.12±16.90	73.00±7.38	70.34±10.01
***2^nd^ Semester***									
Theory	52.50±1.024	42.08±28.87	62.59±17.40	59.42±18.88	54.64±6.51	64.72±17.94	72.04±9.19	58.33±24.57	66.80±8.21
Practical	66.00±13.31	47.32±32.49	65.79±18.73	57.573±18.69	66.23±8.72	76.35±7.63	79.92±5.73	70.55±27.34	67.10±16.72
Grand Total	59.23±12.36	44.75±30.64	63.29±17.26	58.57±19.15	64.11±7.42	65.26±18.13	75.64±7.23	63.66±25.44	71.14±7.59
***3^rd^ Semester***									
Theory	63.34±12.13	58.88±16.28	62.73±20.45	55.42±27.41	42.85±30.39	54.60±8.06	65.40±20.50	53.77±30.80	71.03±5.89
Practical	65.35±12.37	65.39±18.26	66.10±21.14	54.76±27.05	46.00±32.68	63.92±7.62	76.88±23.69	59.00±33.81	77.38±6.09
Grand Total	64.41±12.04	62.20±17.18	64.42±20.88	55.00±27.07	44.50±31.55	59.26±7.49	70.48±21.81	56.11±32.13	73.85±5.29
***4^th^ Semester***									
Theory	58.42±14.91	59.35±23.01	62.35±24.10	49.68±29.03	57.26±26.64	55.34±26.17	69.00±15.96	53.77±30.81	68.75±18.02
Practical	63.51±16.29	63.90±24.46	67.45±24.88	53.39±30.57	58.61±25.09	62.95±29.72	81.36±17.44	65.11±37.05	79.35±19.64
Grand Total	61.01±15.4	61.76±23.70	64.90±24.34	51.47±29.65	57.91±24.81	59.15±27.85	74.44±16.43	58.77±33.51	66.30±28.24

The correlation of semester results with the entrance scores shows (Table-II, III and IV) that in the MBBS program the highest correlation coefficient *r_s_*=0.362 was observed in the second semester of cohort 1 whereas in subsequent cohorts (2 and 3) the highest correlation coefficients (*r_s_*=0.297 and 0.358) respectively were seen in the first semester. In BDS the first semester showed the highest correlation *r_s_* = 0.396, 0.515 and 0.651 in the cohorts (1-3 respectively). In Pharm-D significant correlation was seen in all the four semesters of cohort 1 but not in the subsequent cohorts. The correlation coefficients obtained in this study from all three programs ranged from *r_s_* = 0.213-0.714.

**Table-II:** Correlation of Semester results with entrance test scores MBBS.

	Cohort	Semester score		

		Theory	Practical	Grand Total
Entrance Test Score	1	***1^st^ Semester***		
		0.241*	0.352**	0.312**
		***2^nd^ Semester***		
		0.367**	0.329**	0.362**
		***3^rd^ Semester***		
		0.309**	0.284**	0.329**
		***4^th^ Semester***		
		0.296**	0.255*	0.280*
	2	***1^st^ Semester***		
		0.334**	0.274**	0.297**
		***2^nd^ Semester***		
		0.147	0.140	0.160
		***3^rd^ Semester***		
		0.208*	0.099	0.160
		***4^th^ Semester***		
		0.134	0.063	0.111
	3	***1^st^ Semester***		
		0.334**	0.361**	0.358**
		***2^nd^ Semester***		
		0.277**	0.321**	0.302**
		***3^rd^ Semester***		
		0.266**	0.284**	0.268**
		***4^th^ Semester***		
		0.208*	0.244*	0.213*

** Correlation is significant at the 0.01 level,

* Correlation is significant at the 0.05 level.

## DISCUSSION

The admission criteria has a critical impact on the students’ future and on the quality of output of the education system. The test-criterion validity evidence for admission test is immensely valuable and determined by correlating the scores from the admission test with the outcome variable of interest.^4^

On the subject of interpreting correlation coefficients Tayler considers values of correlation (*r*) less than 0.35 showing a weak correlation.^22^ However, Kelly et al., reported criterion related validity which identifies that coefficients greater than r = 0.5 are rare and values in range of r = 0.2-0.29; although low; may be of statistical significance.^23^ For MCAT predictive validity coefficients of r=0.4 or greater are indicative of a strong relationship.^10^

The present study shows that the spearman rank correlation coefficient revealed statistically relevant; weak to moderately positive associations between the entrance test and the in-course performance in the three selected programs, with stronger and consistent correlation both in MBBS and BDS which was higher in semester one and lower in subsequent semesters. For each cohort 1, 2 and 3, inconsistent correlation was observed in Pharm-D program, where significant correlations were observed only in cohort 1.

In the discipline of medicine (MBBS) the entrance test was found to be more strongly correlated with academic performance in the first and the second semesters (*r_s_* = 0.358, 0.362 respectively) as compared to the third and fourth *(r_s_* = 0.268, 0.213 respectively) semesters. Comparing studies on the predictive validity of MCAT have shown correlations between MCAT and academic performance between 0.3-0.6 9,10 Similarly, the Saudi health science and medical schools preadmission tools have reported strong correlation between academic performance measured by GPA of first six semesters and aptitude exams in year one and two of 2008 and 2009 (r = 0.81 and 0.78) respectively and then a decline in the strength of association (r = 0.74 and 0.66) in semester one and two of cohort 1.^6^

In this study a similar decline in strength of association is also observed for the BDS program with higher correlations for the first semester (*r_s_*= 0.396, 0.515 & 0.651) as compared to semester three and four (*r_s_* =0.478 & 0.427 respectively). This may be similar to the findings of Sandow et al. who report that the DAT scores are statistically significant for academic performance in year one, two and three; with the highest value of *r* = 0.475 in year one decreasing to 0.348 in year two and *r* = 0.129 in year three. 12

Correlation of entrance test score with academic performance in Pharmacy shows a different pattern. A decline in strength of relationship is not noted as a student progresses in the academic years and significant values are seen only in cohort 1 with higher values for the fourth semester (*r_s_* = 0.704) as compared to the first semester (*r_s_* = 0.682). This may be attributable to change in pattern of test in subsequent years and non-availability of data set of some students which led to less number of students from pharmacy as compared to medicine and dentistry. PCAT has been found to establish moderate to strong correlation with pharmacy program grades.^24^

**Table-III:** Correlation between Semester results with entrance test scores BDS.

	Cohort	Semester score		

		Theory	Practical	Grand Total
Entrance Test Score	1	***1^st^ Semester***		
		0.320	0.414*	0.396*
		***2^nd^ Semester***		
		0.223	0.121	0.176
		***3^rd^ Semester***		
		0.064	0.177	0.123
		***4^th^ Semester***		
		0.046	0.119	0.080
	2	***1^st^ Semester***		
		0.464**	0.527**	0.515**
		***2^nd^ Semester***		
		0.415*	0.541**	0.504**
		***3^rd^ Semester***		
		0.494**	0.457**	0.486**
		***4^th^ Semester***		
		0.365*	0.393*	0.427*
	3	***1^st^ Semester***		
		0.651**	0.619**	0.651**
		***2^nd^ Semester***		
		0.263	0.239	0.270
		***3^rd^ Semester***		
		0.441**	0.456**	0.478**
		***4^th^ Semester***		
		-0.001	0.155	0.126

** Correlation is significant at the 0.01 level,

* Correlation is significant at the 0.05 level.

**Table-IV:** Correlation between Semester score with entrance test score Pharm-D.

	Cohort	Semester score		

		Theory	Practical	Grand Total
Entrance Test Score	1	***1^st^ Semester***		
		0.585**	0.615**	0.682**
		***2^nd^ Semester***		
		0.631**	0.0643**	0.652**
		***3^rd^ Semester***		
		0.531**	0.542**	0.571**
		***4^th^ Semester***		
		0.657**	0.600**	0.704**
	2	***1^st^ Semester***		
		0.098	0.085	-0.127
		***2^nd^ Semester***		
		-0.127	-0.185	-0.169
		***3^rd^ Semester***		
		-0.061	-0.104	-0.104
		***4^th^ Semester***		
		-0.509	-0.335	-0.460
	3	***1^st^ Semester***		
		0.145	0.050	0.150
		***2^nd^ Semester***		
		0.001	-0.186	-0.078
		***3^rd^ Semester***		
		-0.147	-0.092	-0.132
		***4^th^ Semester***		
		0.106	-0.030	0.121

** Correlation is significant at the 0.01 level,

*Correlation is significant at the 0.05 level.

Internationally as well in Pakistan, no uniform decision is drawn with respect to the predictive validity of the MCAT, DAT and PCAT. It is unsettled if the admissions test truly predict pre-clinical or clinical performance as both weak to moderately high values (correlation coefficients) are reported in studies both from; within Pakistan and from other countries.

The results of this study indicate that the Ziauddin University’s ‘uniform entrance test’ has weak to moderate predictive validity for performance in the first two years of health professional students of medicine and dentistry graduate level programs. As the UET is not an equal predictor for the three selected programs; therefore there is need for the admission decision makers to review the entrance test for appropriateness to all health science programs. While all the three colleges employ the same entrance test for selection of the students, the criterion variable; the in-course performance assessment is different, both theory and performance based are held but the courses differ significantly particularly MBBS and BDS are significantly different from the Pharm-D program.

Ali et al., (2017) studied different components of the Aga Khan University MCAT (AKU-MCAT) and identified that sub test scores in English, Mathematics and Biology predicted overall academic achievement in medical school; while scores in Chemistry and Physics had low validity coefficients.^25^ Therefore it may be considered to increase weightages of English, Mathematics and biology and undertake further studies to determine desirable weightages of different components in different programs.

It is concluded, that better performance in the entrance test predicts higher semester scores and more likelihood of achieving higher scores in the first year as compared to the subsequent years. From the results it is evident that the uniform entrance test at ZU is more predictive of performance in MBBS and BDS than in Pharm-D.

Future studies can be undertaken to determine which component of the admission test is best at predicting future performance in a selected discipline; the results of which can then guide allocation of appropriate weightage to the different components for the different programs.
